# The role of public-private partnerships in extending public healthcare provision to irregular migrants: stopgap or foot in the door?

**DOI:** 10.1186/s13584-020-00406-0

**Published:** 2020-09-24

**Authors:** Nora Gottlieb, Dani Filc, Nadav Davidovitch

**Affiliations:** 1 Department of Health Care Management, Berlin Technical University, Berlin, Germany; 2 Department of Population Medicine and Health Services Research, Bielefeld School of Public Health, P.O. Box 100131, 33501 Bielefeld, Germany; 3 Department of Politics and Government, Ben-Gurion University of the Negev, Beersheba, Israel; 4 Department of Health Systems Management, Ben-Gurion University of the Negev, Beersheba, Israel

## Abstract

In this commentary to the paper “Ensuring HIV care to undocumented migrants in Israel: a public-private partnership case study” by Chemtob et al. we discuss the role of public-private partnerships (PPPs) as a mechanism for integrating previously excluded groups in public healthcare provision. Drawing on PPP case-studies as well as on Israel’s pandemic preparedness policies during the Covid-19 outbreak, we examine potential implications for the populations in question and for health systems.

In our view, Chemtob et al. describe an exceptional achievement, where a PPP served as a stepping stone for the subsequent integration of irregular migrants’ in publicly funded HIV care. However, we argue that in many other cases PPPs are liable to undermine public healthcare and inclusionary claims. This view is informed by the fundamentally different concepts of healthcare that underlie PPPs and public healthcare provision (namely, health care as a commodity vs. access to healthcare as a right) and existing evidence on PPPs’ role in facilitating welfare retrenchment. In contexts that are dominated by an exclusionary stance toward irregular migrants, such as contemporary Israel, we believe that PPPs will become stopgaps that undermine health rights, rather than a first foot in the door that leads toward equitable provision of healthcare for all.

## Introduction

In many welfare states, so-called “irregular migrants” remain excluded from public healthcare provision, despite governments’ commitments to universal health coverage and the Sustainable Development Goals’ pledge to “leave no one behind” [1]. “Irregular migration” denotes human mobility outside those migration channels that are foreseen and authorized by states, such as bilateral labour migration arrangements. Being an “irregular migrant” is thus the outcome of interrelations between human movement across social spaces and states' enactment of policies within the same spaces [2].

The question of irregular migrants’ access to public healthcare epitomizes a tension inherent to the national welfare state-concept: On the one hand, the welfare state is an instrument for the realization of social and health rights and inclusion. On the other hand, it safeguards the nation’s public resources by distinguishing between the “deserving” and the “undeserving”, for example, along the lines of national citizenship, and by excluding the latter from benefits [3].

The Covid-19 pandemic reminds us, however, that health in a globalized world transcends national frameworks. Exposure to hazardous living and working conditions and exclusion from health services jeopardizes not only the health and lives of the excluded, but of all. Therefore, several states have included irregular migrants in their Covid-19 response. For example, in the UK, uninsured migrants diagnosed with Covid-19 are exempt from charges for medical treatment (to which the NHS visitors and migrant cost recovery programme would otherwise apply) [4]. The Berlin Senate has established a temporary arrangement for anonymous and gratis ambulatory care for uninsured migrants [5, 6]. Portugal went so far as to endow all migrants with temporary citizenship, including eligibility for the National Health Services [7]. The Israeli government stated that there was “no choice” but to expand preparedness measures to irregular migrants [8]. It thus reaffirmed its exclusionary stance, while expressing the exigence to provide Covid-19-related healthcare for all [9]. The Covid-19 pandemic is an exceptional situation. But as such it helps understand “the normal”.

Governments always have a choice whether or not to include formerly excluded groups in public healthcare provision. And they have a choice regarding the mechanism of inclusion. This raises several interrelated questions, which ultimately touch on our understanding of what public health is: Which situations compel governments to expand public healthcare provision? What mechanisms do they choose? And what are the implications? In this commentary on the paper “Ensuring HIV care to undocumented migrants in Israel: a public-private partnership case study” [10] by Chemtob et al., we discuss the implications of public-private partnerships (PPPs) as a mechanism of inclusion for the population in question as well as for the respective health system. We also briefly relate to rationales for expanding public healthcare provision.

## Three arguments against public-private partnerships as a means to expand public healthcare

Chemtob et al. describe a PPP that was established to provide HIV treatment for undocumented migrants in Israel. They note that “this is the first example of a PPP with state partnership in a high-income country to address an extreme need among the undocumented community” [10, p. 8]. They further describe as the initiative’s major success that the service was eventually integrated into the Israeli healthcare system, with costs covered by the Ministry of Health.

We could not agree more about the idea that the initiative merits praise for obtaining universal HIV treatment from the Israeli government. What makes this an exceptional accomplishment is that it expanded coverage in the most unlikely political context; namely, to undocumented migrants and thus to a population for whose health needs the Israeli government does not usually consider itself responsible [11, 12].

However, can this PPP case-study be a role-model for other populations and for other health needs? Are PPPs a desirable strategy for extending public healthcare provision to marginalized groups? Can they be a first “foot in the door” to be incrementally turned into inclusion? We think that the answer is “no”, the success of this case notwithstanding.

As mechanisms for delivering health services, public healthcare provision and PPPs reify fundamentally different conceptualizations of healthcare: The welfare state provides healthcare as a right. PPPs provide healthcare as a commodity. While public participation in PPPs aims to assure universal access, the involvement of for-profit organizations in the provision of services make market considerations the dominant variable in decision-making - with far-reaching implications: First, for-profit organizations will prioritize profits over issues of accessibility, quality and patient empowerment. Second, understanding healthcare as a commodity essentially influences the approach to public health issues by narrowing public health down to the delivery of (mainly biomedical) services that can be quantified and priced; for instance, vaccinations or screening. Such commodification of public health practices, however, does not allow addressing the social determinants of health; because that requires intersectoral action on population level, which cannot be easily quantified and priced. Third, it is the exception and not the rule that a service that began as a PPP is subsequently transformed into a state responsibility.

Examples of PPPs worldwide - such as the British Private Finance Initiative [13] and the Spanish Alzira experiment [14, 15] - suggest that PPPs are in fact often a form of subtle privatization of healthcare. In most cases, they fill voids where welfare states retreat from responsibilities. PPPs alleviate the worst impacts of cutbacks in public healthcare provision in the short term. In the long run, they normalize and solidify privatization by making market considerations the central criteria for decisions, for example, over which types of health services to develop (such as the expansion of services aimed at lucrative “market shares” like, for example, vaccines for travellers) and over their geographical distribution (for instance, concentration in high-income areas versus equitable access across central and peripheral regions). Eventually, PPPs are thus liable to expedite the erosion of public healthcare systems [16–18]. The risk of PPPs facilitating neoliberal welfare retrenchment has been described for the Israeli case [19, 20]. Hence, in those cases in which PPPs emerge as stopgaps for public healthcare provision, they may undermine the concept of health as a universal and indivisible right, rather than help realize health rights.

## Questioning the rationale for public healthcare expansion: “protection of” or “protection from”?

The primary rationale for providing treatment to a population that otherwise remains barred from public healthcare, both in the case of HIV and Covid-19, is apparently fear of contagion. The logic that works for the success of Chemtob’s et al. case-study of HIV treatment for undocumented migrants is the same “Us vs. Them”-logic that undergirds the exclusion of this population. The state provides treatment when indispensable to protect “us” from the risks “they” embody [21, 22] – and only then. Without the risk of contagion (for example, in case of non-communicable diseases or injuries), the same logic does not work. Moreover, from an “Us vs. Them”-perspective, governments will favour solutions that allow providing just enough healthcare to reduce risks to the majority population, while avoiding challenges to the welfare state’s boundaries. PPPs offer a convenient way to add on solutions that provide excluded populations with a minimum level of healthcare, instead of making systemic changes toward universal health coverage and the highest attainable standard of health (also) for migrants, as is advised, inter alia, by the WHO constitution, the International Covenant on Economic, Social and Cultural Rights, and the recent WHO action plan “Promoting the health of refugees and migrants” [23]. We claim that PPPs, in most cases, will not work as stepping stones toward these visions. The PPP-inherent commodification of healthcare, in combination with an “Us vs. Them”-logic, is liable to generate a very limited conception of public health. Instead of a holistic approach that addresses the social determinants of health, entrenched in an ethics of solidarity, social justice, and human rights, public health is conceived as a set of interventions that ensure “protection from”, rather than “protection of”. Within such conceptual framework, the health of marginalized migrants is a means, not the goal.

In the Israeli context, the latest manifestation of such a stance is the government’s response to the Covid-19 pandemic. In a laudable move, it decided to provide free testing and treatment for all irregular migrants. However, in a context where the Covid-19-related lockdown left many irregular migrants without any income and pushed families into absolute destitution, a broader perspective of public health would have required taking into consideration also the social determinants of health such as livelihoods. The “Deposit Law” offered an easy way out: By this law, 20% of asylum-seekers’ wages are deducted as a deposit, payable upon their departure from Israel. Local and international agencies (including the UNHCR) urged the government to disburse the retained amounts to needy households as an economic relief. However, the government disbursed the deposits only after the Supreme Court’s intervention [9]. Beyond the duration of the outbreak and beyond Covid-19-related treatment, irregular migrants remain without access to many of the social determinants of health. The patchy inclusion necessitated by the pandemic must therefore be understood as the exception that proves the rule: a tenaciously exclusionary approach toward irregular migrants [9].

## Conclusions

Privatization, welfare retrenchment and ethno-national exclusion form the backdrop against which migrants’ health rights are claimed, contested and re-negotiated in Israel. In this context, we caution against using PPPs as a strategy for extending healthcare provision to marginalized groups. Even though it resulted in inclusion in Chemtob’s et al. case-study, in many other cases PPPs are fraught with the risk to become stopgaps that undermine health rights, rather than a first foot in the door that leads toward equitable provision of healthcare for all. Hence, when considering expanding public healthcare provision to previously excluded groups, policymakers ought to be aware that the seemingly technical choice of a mechanisms for inclusion has far-reaching consequences, for the group in question, for the health system, and the public. As the Covid-19 pandemic requires policymakers to formulate and revise public health policies and solutions - including access to healthcare, testing and other interventions for a variety of different groups - in a rush, such awareness will be all the more important to avoid eroding public health systems from the margins.

## Data Availability

Not applicable.

## Abbreviations

Covid-19: Disease caused by the SARS-CoV-2 (2019-nCoV) coronavirus
HIV: Human immunodeficiency virus
PPP: Public-private partnership
UNHCR: Office of the United Nations High Commissioner for Refugees
WHO: World Health Organization
